# Identifying and Prioritizing Age-Friendly Design Principles and Guidelines for Developing Transportation Planning E-Tools: Scoping Review

**DOI:** 10.2196/83668

**Published:** 2026-03-19

**Authors:** Sara Bahrampoor Givi, Mireille Gagnon-Roy, Véronique Provencher

**Affiliations:** 1 School of Rehabilitation, Faculty of Medicine and Health Sciences, Université de Sherbrooke, 3001, 12e Avenue Nord, Sherbrooke, QC, J1H 5N4, Canada, 1 8198218000

**Keywords:** older adults, usability, guidelines, review, transportation, e-tools

## Abstract

**Background:**

Older adults often face mobility challenges and usability barriers when navigating transportation options due to age-related physical, cognitive, and sensory changes. While transportation planning e-tools can support their independence, most are not designed for their specific needs. There is a lack of comprehensive, age-friendly usability design principles tailored to this context.

**Objective:**

This study aims to identify, synthesize, and prioritize the most relevant age-friendly usability design principles and guidelines for developing transportation planning e-tools that are tailored to the needs of older adults.

**Methods:**

A scoping review was conducted following the Arksey and O’Malley methodological framework, enhanced by guidance from the Joanna Briggs Institute and reported according to the PRISMA-ScR (Preferred Reporting Items for Systematic Reviews and Meta-Analyses Extension for Scoping Reviews) standards. The literature search was performed across the following six scientific databases: MEDLINE (PubMed and National Center for Biotechnology Information), AgeLine (EBSCOhost), Cochrane Library (Wiley Online Library), Scopus (Elsevier), IEEE Xplore, and TRID (Transportation Research Board), and was supplemented by gray literature identified through Google Scholar and Google Search, covering the period from January 2013 to April 2025. To guide the analysis, 4 foundational usability frameworks by Nielsen; Shneiderman and Plaisant; Gerhardt-Powals; and Weinschenk and Barker were used to inductively derive generic principles that structured the classification of age-friendly guidelines. This process resulted in the organization of extracted guidelines into 10 core usability principles. The analytic hierarchy process was applied by a single expert to rank the principles based on expert pairwise comparisons and their frequency of reference in literature.

**Results:**

The study identified 31 relevant studies. From these, 500 age-friendly guidelines were refined into 68 actionable guidelines across 10 usability principles: visual clarity, structure and navigation, ease of use, information, minimizing memory load, feedback, accessibility, consistency, simplicity, and control. The analytic hierarchy process ranked visual clarity (36.4%), structure and navigation (22.1%), and ease of use (12.5%) as the top 3 age-friendly design priorities.

**Conclusions:**

This study offers an evidence-based foundation for developing transportation planning e-tools that promote older adults’ autonomy and digital inclusion, with prioritized guidelines applicable to transportation planning e-tools and to the broader field of age-friendly digital design. Ongoing updates and active user involvement are essential to ensure their sustained usability and long-term relevance.

## Introduction

### Background

As people age, they often encounter mobility-related difficulties, which can significantly impact their ability to engage in society [1]. These difficulties can make everyday activities, such as attending medical appointments or participating in social and recreational events, increasingly challenging [2]. Reduced physical capacity, changes in vision [3] and balance [4], as well as the onset of chronic illnesses [4], contribute to these mobility issues. As a result, many older adults find it difficult to maintain the independence and social connections that are crucial to their overall well-being [5]. Addressing these challenges requires a comprehensive approach, including accessible transportation options that help older adults remain active and engaged in their daily lives, despite steep hills and heavy snowfall, which are common in Nordic countries like Canada.

In the province of Quebec (Canada), data from 2019 show that approximately 64% of older adults drive and 23% travel as passengers, while about 9% use taxis, adapted transport, or public transit, and only 3% rely on walking, cycling, or mobility devices. Men were more likely than women to drive (80% vs 50%), while women more often traveled as passengers (35% vs 10%) and used taxis, paratransit, or public transit more frequently (12% vs 7%) [6]. Although only a small proportion of older adults use public or shared transportation, studies indicate that this limited use does not necessarily reflect a lack of interest but rather barriers such as safety concerns, difficulties accessing clear travel information, and a lack of confidence or comfort when using available options like buses—often due to fears of becoming disoriented or encountering unwanted social interactions [7,8].

To mitigate these challenges, there is a growing need for transportation planning e-tools that can assist older adults in accessing reliable information about their travel options. E-tools (eg, route-planning systems) could help drivers feel safer by avoiding hazardous routes, such as steep hills or poorly lit roads, particularly in challenging conditions [9]. However, engaging with such digital tools often proves difficult for this population. Age-related changes such as reduced visual sensitivity [10] can make it hard to accurately select interactive elements, thereby disrupting the overall trip-planning experience [11]. In addition, cognitive factors, such as reduced attention, can make it more difficult to navigate digital interfaces [12]. Consequently, the design of transportation planning e-tools must consider both the functional changes associated with aging and the diversity in digital competencies among older adults [13]. It is therefore essential to adapt these tools, particularly their interfaces, to better meet the needs of older adults.

Given the notable lack of studies specifically focused on developing and evaluating transport planning interfaces tailored to the needs and preferences of older adults through a participatory design approach [14], it becomes increasingly important to identify comprehensive age-friendly design principles and usability guidelines. While a number of design frameworks exist for general usability [15], few explicitly address the cognitive, physical, and perceptual changes associated with aging [16,17]. Without clear, context-specific design guidance, there is a risk that digital solutions will continue to fall short of accessibility and usability standards for this population. A systematic synthesis of existing guidelines is relevant to ensure that future e-tool interfaces are not only inclusive but also practical, empowering older adults to confidently plan and manage their trips. It is therefore essential to fill the gap by identifying, consolidating, and prioritizing existing age-friendly usability design principles to inform the development of transportation planning e-tools that are both effective and attuned to the diverse capabilities of older users.

### Objective

This study aims to identify, synthesize, and prioritize the most relevant age-friendly usability design principles and guidelines for developing transportation planning e-tools that are tailored to the needs of older adults.

## Methods

### Study Design

This scoping review was conducted using the methodological framework proposed by Arksey and O’Malley [18], which includes five stages: (1) identifying the research question; (2) identifying relevant sources; (3) selecting studies; (4) charting the data; and (5) collating, summarizing, and reporting the results. Methodological guidance from the Joanna Briggs Institute was also integrated to enhance the rigor of the review. The reporting process adheres to the PRISMA-ScR (Preferred Reporting Items for Systematic Reviews and Meta-Analyses Extension for Scoping Reviews) guidelines (Checklist 1) [19]. In the second phase, the analytic hierarchy process (AHP) [20] was used to prioritize the age-friendly usability design principles identified through the scoping review.

### Scoping Review: Identifying the Research Question

The study is guided by the following research question: What are the most relevant age-friendly usability design principles and guidelines for developing transportation planning e-tools tailored to the needs of older adults? This question was structured using the population, concept, and context (PCC) framework, where the population is older adults, the concept focuses on age-friendly usability design principles and guidelines, and the context involves the development of transportation planning e-tools.

### Identifying the Relevant Sources

This stage consisted of searching scientific and gray literature published in English from January 2013 to May 2023, with an updated search conducted twice, in April 2024 and April 2025. This timeframe was selected to capture the most recent decade of research on age-friendly usability guidelines and transportation planning e-tools, reflecting rapid advances in digital technologies. The search was performed across six (6) scientific databases: MEDLINE (PubMed and National Center for Biotechnology Information), AgeLine (EBSCOhost), Cochrane Library (Wiley Online Library), Scopus (Elsevier), IEEE Xplore, and TRID (Transportation Research Board). To supplement the scientific literature, gray literature was identified through Google Scholar and Google Search, with the first 50 results from each platform screened for inclusion to ensure relevance and manageability.

The development of the search strategy followed a systematic and collaborative approach. Initially, authors compiled a list of relevant databases and preliminary keywords based on the study objectives. This preliminary version was then critically reviewed by a professional librarian, who contributed methodological expertise throughout the process. His recommendations were informed by the PRESS (Peer Review of Electronic Search Strategies) 2015 guidelines [21], leading to substantial improvements in keyword formulation, the use of Boolean operators, and the integration of controlled vocabulary terms. To improve the effectiveness of the search strategy, the professional librarian suggested running preliminary tests to evaluate how well the selected terms captured relevant studies. Based on the outcomes, he recommended several refinements. For example, the terms “Graphical User Interface” and “GUI” were added to ensure that studies using different terminology could be retrieved. These adjustments aimed to reduce the number of irrelevant results and to ensure that key studies were not overlooked.

Controlled vocabulary, such as Medical Subject Headings terms in PubMed, was mapped and complemented by a tailored list of free-text terms to reflect diverse indexing practices and author phrasing across databases. After executing the search, the results were imported into Zotero (Corporation for Digital Scholarship) for reference management. Initial deduplication was performed using Zotero’s automated function, followed by a thorough manual screening by the first author (SBG) to remove any remaining duplicate records. The final search strategy, including both controlled vocabulary and keywords, is presented in Table 1.

**Table 1.** Keywords and concepts.

Concepts	Keywords	Controlled vocabulary (by database)
Aging	“Older adult” OR “Old people” OR “Elderly” OR “Senior” OR “Aging” OR “Normal aging” OR “Frailty” OR “Aged” OR Old*	MEDLINE or Cochrane Library: aged (MeSH^a^) AgeLine: aged, older adults TRID: elderly persons (thesaurus)
E-tool	“Website” OR “Application” OR “E-tool” OR “Web application” OR “App” OR “Web”	MEDLINE or Cochrane Library: mobile applications (MeSH) IEEE Xplore: software applications (IEEE thesaurus) TRID: websites (TRID thesaurus)
Guideline	“Design principles” OR “Web design principle” OR “Design guideline” OR “Web design guideline” OR “Usability guideline” OR “User interface design guideline” OR “UI design guideline” OR “User experience design guideline” OR “UX design guideline” OR “GUI” OR “Graphical user interface”	MEDLINE or Cochrane Library: user-computer interface (MeSH) and guidelines as topic (MeSH) AgeLine: human-computer interaction and usability engineering TRID: Human factors engineering
Transport	“transport* OR “public transport*” OR mobility* OR travel OR “public transit” OR “active transport*” OR “alternative transport” OR paratransit OR bus* OR carpool*	MEDLINE or Cochrane Library: transportation (MeSH) AgeLine: transportation TRID: transit, mobility, and public transportation

a MeSH: medical subject headings.

### Selecting the Studies

To be eligible for inclusion, sources needed to address usability guidelines specifically aimed at older adults.

Sources were excluded if they met any of the following criteria: (1) written in a language other than English and (2) focused on e-tools other than websites or apps.

The first author (SBG) conducted the initial screening of all identified sources based on titles and abstracts, followed by a full-text review to assess their eligibility. To mitigate potential bias and strengthen the reliability of the process, 20% of the study selection was independently reviewed and validated by a co-author (VP). Specifically, the co-author independently reviewed 20% of the records retained after the title and abstract screening stage, as well as 20% of the studies assessed at the full-text eligibility stage, to verify inclusion decisions and ensure consistency.

### Charting the Data

To chart the data, 4 pioneering sets of general usability guidelines were selected: Nielsen [15], Shneiderman and Plaisant [22], Gerhardt-Powals [23], and Weinschenk and Barker [24]. These foundational works have stood the test of time, are widely cited in the fields of human-computer interaction and user experience, and have been extensively validated across diverse domains and user groups. Each set offers a distinct yet complementary perspective on usability: Nielsen outlined 10 usability heuristics, Shneiderman and Plaisant introduced 8 golden rules of interface design, Gerhardt-Powals proposed 10 cognitive principles for interface development, and Weinschenk and Barker organized their recommendations into 11 thematic categories. It is important to note that these frameworks were identified before screening relevant studies and were not part of the scoping review dataset. Rather, they were used to develop an initial analytical framework (charting form) to guide the systematic extraction and categorization of age-friendly usability guidelines from included studies. This approach ensured that the analysis was structured, consistent, and comprehensive, while preventing selection bias.

To develop the charting form, the first author (SBG) thematically analyzed the 4 pioneering sets of selected general usability guidelines using the Braun and Clarke [25] inductive thematic analysis approach. Guidelines from key authors were compiled into a cross-table and grouped into broader categories based on emerging patterns. Redundant or overlapping items were removed or integrated to ensure clarity and uniqueness. These refined categories were then defined as 10 core usability principles associated with 19 generic usability guidelines (Table 2).

**Table 2.** Interface design principles with associated generic guidelines.

Principles and guidelines		Shneiderman	Nielsen	Weinschenk and Barker	Gerhardt‐Powals
Feedback					
Informative feedback		✓	✓	✓	
Confirmation of the actions that are done successfully		✓		✓	✓
Let users be aware of results				✓	
Consistency					
Present new information in familiar frameworks and structure					✓
Consistency in using familiar icons and colors		✓	✓		✓
Minimizing the memory load					
Memory load avoidance		✓	✓		✓
Avoid changes in familiar behavior to speed up the action of the experienced users		✓			
Simplicity					
Simple error message (not codes)		✓	✓	✓	
Simple and relevant information (eliminate unnecessary information to reduce the use of the resource in multitasks)			✓		✓
Simple layout				✓	✓
Accessibility					
Consider user profile in design (eg, digital literacy)		✓	✓		
Flexibility in providing information for a wide range of users with different cognitive abilities, in different tasks, for both experienced and novice users			✓		✓
Structure and navigation					
Error prevention by implying target limitation (eg, do not allow the user to enter numeric characters in alphabetic fields)		✓	✓	✓	
Data grouping					✓
Control					
Ability to control by undo, redo, or exit		✓	✓	✓	
Command and control model to let users know how they should answer the questions and enter the information				✓	
Ease of use					
Easy to access help and support			✓		✓
Visual clarity					
Use graphics as much as possible instead of text					✓
Information					
Use names that are conceptually related to function					✓

Subsequently, a structured Microsoft Excel grid was developed to chart the sources identified through this scoping review. In this grid, source characteristics (including publication year and study context), e-tool types, and age-friendly guidelines, categorized according to usability principles derived from the 4 pioneering general usability frameworks mentioned earlier, were captured. Similar to the generic usability design guidelines, the Braun and Clarke [25] thematic analysis framework was used. Since the generic usability guidelines served as the foundation for developing age-friendly usability guidelines, a deductive approach was chosen. In this context, all the identified guidelines were compiled into a cross-table. This process involved reading through the guidelines to understand their content and context, ensuring that each entry was accurately represented in a table. In the next step, each guideline was labeled with a principle that best represented its content, facilitating the organization of the guidelines into manageable pieces for further analysis. Subsequently, guidelines with the same labels were grouped together. The labeled guidelines were then reviewed and refined to ensure accurate clustering. Redundant guidelines within each group were identified and removed to simplify the dataset. Additionally, similar guidelines were integrated to reduce overlap and ensure that each guideline was distinct and relevant. Finally, the process concluded with final reporting. This process resulted in a clear and distinct set of guidelines.

### Collecting, Summarizing, and Reporting Results

A combination of descriptive-analytical methods and thematic analysis [25] was applied to examine all data categories, including source characteristics, e-tool types, and age-friendly guidelines. The findings were then synthesized and interpreted to map the most relevant age-friendly usability design guidelines for creating transportation planning e-tools tailored to older adults’ needs, categorized in the 10 core usability principles previously defined.

### Prioritization Using the AHP

To prioritize the age-friendly design principles identified through the scoping review, the AHP [20] was used. This method enables prioritization through pairwise comparisons, relying on expert judgment to establish a structured hierarchy of priorities [20]. A key advantage of AHP is its systematic approach to comparing and ranking alternatives or criteria [26]. To guide the pairwise comparisons, the Saaty’s [20] fundamental scale of relative importance was used. This scale assigns numeric values to verbal judgments: 1 for equally important, 3 for moderately important, 5 for strongly important, 7 for very strongly important, and 9 for extremely important. The even numbers 2, 4, 6, and 8 serve as intermediate values between these main levels.

To validate the results of the AHP, the consistency ratio (CR) is calculated using the formula CR=CI/RI, where the consistency index (CI), derived from pairwise comparisons, is measured using the following formula and then compared with the corresponding random index (RI).


$$CI=\frac{\lambda_{max}-n}{n-1}$$

In this study, the AHP Online System (AHP-OS), developed by Goepel [27], was used by the first author (SBG), who has extensive experience in human-centered design and usability evaluation, to conduct pairwise comparisons of each design principle. The software aggregated these comparisons and calculated normalized priority weights. Principles with higher frequencies in the scoping review were considered more influential and were assigned higher relative importance values on the Saaty fundamental scale (eg, “moderately” to “very strongly” more important) compared with less frequently cited principles. This approach ensured that the prioritization systematically reflected both the literature evidence and expert judgment. Using this method facilitated decision-making by quantifying expert input, producing a consistent ranking of the most critical age-friendly usability design principles, and resolving conflicts between design criteria.

## Results

### Scoping Review

**Figure 1.**
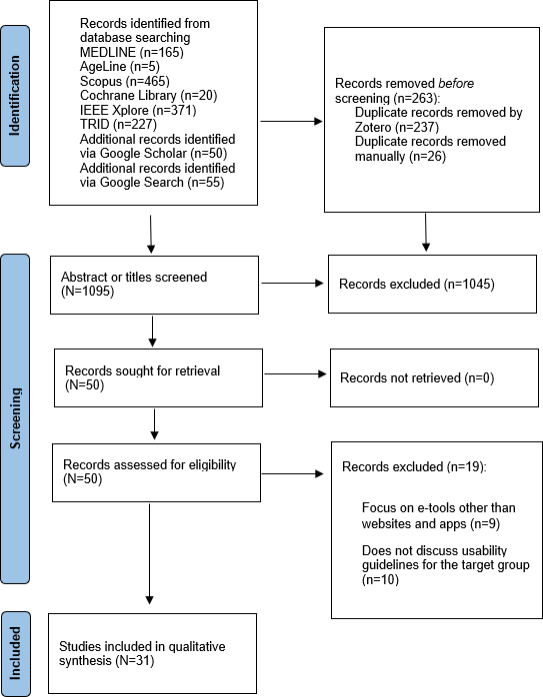
PRISMA (Preferred Reporting Items for Systematic Reviews and Meta-Analyses) 2020 flow diagram detailing the process of literature search, screening, and selection.

A total of 1358 sources were initially identified through database searches and gray literature. After removing duplicates, 1095 unique sources remained. Following the screening of titles and abstracts, 50 sources were retained. Of these, 19 did not meet the inclusion and exclusion criteria, leaving 31 sources for full-text analysis (Figure 1).

### Characteristic of Sources

Out of the 31 sources (Table 3), 26 (83.9%) clearly indicated the publication year, ranging from 2001 to 2024 [28-53]. Most (n=24, 77.4% publications) guidelines were published between 2015 and 2024 [28-30,32-44,46-53], with a peak in 2023 (n=5, 16.1% publications) [29,39,41,44,49].

**Table 3.** List of studies included in the scoping review.

Title	Year	Context of the study	E-tool type	Number of guidelines identified
*Interface Design Features of Mobile Application for Senior Citizens* [28]	2019	Religion	App	3
*UsAge Guidelines: Toward Usable Saudi M-Government Applications for Elderly Users* [29]	2023	Public services	App	22
Android OS [30]	2019	—^a^	App	5
*Design and Evaluation of a Mobile User Interface for Older Adults: Navigation, Interaction and Visual Design Recommendations* [31]	2014	Gaming	App	8
*Designing Digital Games With & for Home-Dwelling Older Adults” Social Interaction Under Sheltering Measures* [32]	2021	Gaming	App	2
*UX^b^/UI^c^ Design for Elderly Users: Best Practices & Principles* [33]	2024	—	—	9
*Guideline for Web Pages Design Inclusive for Senior Users* [34]	2021	—	Website	56
*Designing for an Aging Population: Toward Universal Design* [35]	2016	—	Website	40
*A Guide to Designing for Older Adults* [36]	2024	—	—	9
*Examining the Usability of Touch Screen Gestures for Older and Younger Adults* [37]	2015	—	App	5
*Towards Accessibility Guidelines of Interaction and User Interface Design for Alzheimer’s Disease Patients* [38]	2017	—	App	121
*Design Guidelines of Mobile Apps for Older Adults: Systematic Review and Thematic Analysis* [39]	2023	—	App	19
*UX/UI Design for Elderly Users: A Comprehensive Guide* [40]	2024	—	—	3
*Age Inclusive Digital Platforms* [41]	2023	—	—	17
*Development of a Web-Based Insulin Decision Aid for the Elderly: Usability Barriers and Guidelines* [42]	2017	Health	Website	25
*Mobile Health Apps: Improving Usability for Older Adult Users* [43]	2019	Health	App	19
*Developing Usability Guidelines for mHealth Applications (UGmHA)* [44]	2023	Health	App	1
*Making Your Website Senior Friendly: A Checklist* [45]	2001	—	Website	22
*Increasing Usability of Homecare Applications for Older Adults: A Case Study* [46]	2019	Health	App	5
*Mobile Accessibility: How WCAG 2.0 and Other W3C/WAI Guidelines Apply to Mobile* [47]	2015	—	App	16
*Design of Mobile Phones for Older Adults: An Empirical Analysis of Design Guidelines and Checklists for Feature Phones and Smartphones* [48]	2018	—	App	19
*Evaluating User Interface and User Experience in Mobile Applications Designed for the Elderly* [49]	2023	Health	App	7
*Development of Universal Design Mobile Interface Guidelines (UDMIG) for Aging Population* [50]	2016	—	App	5
*User Interface on Smartphone for Elderly Users* [51]	2017	—	App	5
*A Preliminary Study on Designing Tour Website for Older People* [52]	2015	Tourism	Website	14
*Improving Messenger Accessibility for Elderly Users using User Centered Design (UCD) Methods (Study Case: WhatsApp)* [53]	2021	Communication	App	5
*Age Before Beauty—A Guide to Interface Design for Older Adults* [54]	—	—	Website or app	9
*A Guide to Interface Design for Older Adults* [55]	—	—	Website or app	12
*Factsheet On SS 618—Guidelines on User Interface Design for Older Adults* [56]	—	—	Website or app	3
*Designing for iOS* [57]	—	—	—	9
*UI Design for Elderly* [58]	—	—	—	5

a Data not available.

b UX: user experience.

c UI: user interface.

Of the 31 sources, 11 (35.5%) specified the context of the study. Most of these contexts were related to health, representing 16.1% of the total sources [42-44,46,49]. Other identified contexts included tourism [52], communication [53], gaming [31,32], public service [29], and religion [28].

The majority of sources (n=17, 55%) focused on age-friendly usability guidelines related to developing apps, reflecting the growing use of smartphones and mobile technology among older adults [28-32,37-39,43,44,46-51,53]. Five (16.1%) sources focused on guidelines for developing websites [34,35,42,45,52], and 3 (9.6%) sources covered both websites and apps [54-56]. Additionally, 6 (19.3%) sources did not indicate the type of e-tool [33,36,40,41,57,58].

A total of 500 guidelines were identified. Among them, visual clarity accounted for 26.6% (133 guidelines) and had the highest frequency of referral, while consistency, simplicity, and control each represented 5% (25 guidelines) and had the lowest frequency of referral. For more detailed information on the frequency of referral for each principle, see Table 4.

**Table 4.** Frequency of referral to guidelines associated with each principle.

Principles	Frequency of referral, n (%)
Visual clarity	133 (26.6)
Structure and navigation	82 (16.4)
Ease of use	63 (12.6)
Information	48 (9.6)
Minimizing the memory load	39 (7.8)
Feedback	31 (6.2)
Accessibility	29 (5.8)
Consistency	25 (5)
Simplicity	25 (5)
Control	25 (5)

After removing or integrating redundant or overlapping items to ensure clarity, 74 age-friendly usability design guidelines were identified and categorized under 10 core principles derived from general usability guidelines. The next step involved combining the guidelines identified through the generic usability design guidelines (19 guidelines) with the age-friendly usability design guidelines (74 guidelines), resulting in a total of 93 guidelines. In this process, the guidelines related to each principle were reviewed together, redundant guidelines were removed, and similar ones were combined to reduce overlap. The goal was to ensure that only the most relevant and unique guidelines were retained. As a result, from the initial 93 guidelines, 68 were finalized and categorized into the 10 design principles. Table 5 presents the final set of age-friendly usability design guidelines categorized into 10 core principles.

**Table 5.** Age-friendly usability design principles and guidelines.

Principles and guidelines		Relevant studies
Feedback		
Provide informative feedback by showing clicked links, task status, waiting periods, and error messages with resolution steps.		[15,22-24,29,34,35,38,39,41,48,49,54,57]
Request confirmation before performing actions that could cause significant changes or data loss, and confirm successful actions		[29,33,36,38,41,48]
Consistency		
Maintain consistency in design elements across pages and screens such as by using familiar icons, input formats, colors, menu hierarchy, and so on.		[15,22,23,33-35,38,40,42,43,45,47,57]
Present information clearly by using familiar frameworks and structures, and apply consistent navigation patterns and layouts.		[23,29,38,39,49,50,57]
Underline text only when it is a link.		[58]
Minimizing the memory load		
Present 1 task or process at a time.		[15,22,23,29,34-36,39,47]
Avoid complex dynamic input fields that require multiple gestures or the use of both hands.		[29,42,55]
Eliminate unnecessary or repetitive actions.		[29,38]
Limit the number of key functions on the home screen to 12 or fewer for tablets and 6 or fewer for smartphones.		[38,41,43,46,49,52]
Avoid changes in familiar behavior to speed up the actions of experienced users.		[22,34,35]
Use images, videos, or audio to complement text information.		[38,45]
Minimize time-sensitive content and provide options to adjust or extend time limits if necessary.		[34,35,38,39,41,42,56]
Simplicity		
Simplify data entry processes		[38,43]
Use simple and easy-to-understand graphics.		[43,48,52,55]
Ensure simplicity, clarity, and consistency in screen layout, navigation, and terminology.		[15,23,24,32,34,35,38,39,48,55]
Provide simple error messages (not codes).		[15,22,24,33,36,38,41]
Accessibility		
Consider user profiles in design such as their digital literacy, and visual and cognitive impairment.		[15,22-24,34,38,43,45,47,52]
Ensure button accessibility with a universal approach, accommodating left-handed users.		[34]
Use icons alongside text labels.		[34,38]
Include alternative data entry methods for users unable to use the primary method.		[34,35]
Ensure the app interface adapts to device rotation and user attempts to enlarge screen content.		[29]
Provide content in various multimedia formats, such as text, videos, pictures, and audio.		[30,31,34,35,38,47]
Structure and navigation		
Avoid requiring users to double-click or drag to interact with the page.		[34,36,41,45,50]
Offer both click-to-zoom and 2-finger zoom gestures.		[37,47,48]
Minimize data entry steps to ideally fewer than 3.		[43,52]
Provide help tutorials or brief animated instructions.		[29,33-35,38,46,47,52]
Clearly indicate touchable or clickable elements.		[34,35,39,47,56]
Position key elements (eg, buttons and information) at the center of the screen.		[31,34,35,38,39,41,47,48]
Guide users with systematic navigation to indicate their location and the next step.		[29,34,38,39,41-43,45,52]
Provide a virtual keyboard for numerical or alphabetical entry.		[15,22,24,38]
Use a short, simple, and static pull-down menu.		[34,38,45,48]
Include spell check for errors.		[38]
Maintain hierarchy in feedback, navigation, visual design, and data grouping.		[23,34,35,38,42,47,57]
Avoid using scroll bars, especially horizontally.		[34,35,38,45,48]
Position search tools at the top right.		[34]
Control		
Provide zoom-in ability up to 200%.		[33,38,47]
Consider the home menu as a safe return point.		[31,34,35,43]
Ability to control requests by undoing, redoing, and exiting.		[15,22,24,31,33,36,38,45,57]
Avoid disappearing messages; let users close them manually.		[36]
Offer adjustable text sizes, color schemes, and contrast to enable personalization of the user experience.		[35,38,42]
Ease of use		
Enable a function to remember usernames and passwords.		[34]
Make account creation optional for users.		[34]
Incorporate autosave features whenever possible.		[43,46]
Provide support for both orientations.		[47,57]
Include automatic data entry (eg, time and date) to reduce data entry time and keyboard use.		[29,31,35,38,39,47]
Ensure easy access to help and support.		[15,23,29,35,36,39,41,43,53,55]
Consider target touch areas of at least 9 x 9 mm, with a minimum of 8 dp of inactive space between them.		[30-34,37-39,41-43,47,48,50,54,55,58]
Visual clarity		
Avoid using fluorescent colors and colors with minimal contrast, such as green and red; yellow and blue; or green and blue.		[29,34,35,38,42,43,45,52,54]
Avoid using all capital letters words.		[34,38,45]
Consider 3:1 and 4:1 color contrast ratio for text and large text respectively.		[29,30,34,36,38-40,42,43,48,49,54,55]
Adjust the contrast range between 1.7 and 3.0.		[30,34]
Use blue, red, and magenta as the link colors on a white background due to their high visibility.		[34,36,38,43,45]
Use brightness levels of 75% and 50%.		[34,51]
Use font sizes of 12 point to 14 point for body text and 16 point for titles.		[28,33-35,38-40,43-45,47,48,50-53,55,58]
Use sans serif fonts like Arial, Helvetica, and Tahoma.		[29,34,38,45,51,54,55]
Avoid justified alignment, unfilled text, text shadows, large blocks of italic or bold text, and underlined text.		[38,42,45,54]
Maintain line spacing between 1.5 to 2.		[38,45]
Consider using bold typefaces for emphasis.		[28,38,45,54]
Avoid complex and distracting elements such as animations, blinking text, patterned backgrounds, pure white backgrounds, pure black text on white backgrounds, and transparent menus.		[23,34,38,39,42,44,45,51,57]
Information		
The website should not include pop-up messages or advertisements.		[52]
Avoid unclear or ambiguous language.		[31,33,35,38,39,41,42,45,49,52]
Do not use abbreviations or symbols.		[38]
Ensure each page has a clear and descriptive title.		[38]
Avoid using Roman numerals.		[38]
Use short sentences (less than 70 characters per line) and paragraphs (each focusing on a single idea) and avoid hyphenating words at the end of lines.		[38,39,45,54]
Highlight important information for better visibility.		[34,38]
Use active voice to enhance clarity.		[38,45]
Use names that are conceptually related to function.		[23,34,35,38,42,52,53]

### Analytic Hierarchy Process

AHP was used to assess the relative importance of the 10 identified age-friendly usability design principles. This prioritization was based on expert judgment (SBG) and the frequency of references found in the scoping review. For example, visual clarity had the highest frequency of 133 (26.6%) references, whereas control had 25 (5%) references. During pairwise comparisons, this difference guided the assignment of the Saaty scale values: visual clarity was often judged as “strongly” or “very strongly” more important than principles with lower frequencies. The results indicated that visual clarity, structure and navigation, and ease of use were the most critical principles for the development of transportation planning e-tools tailored to older adults.

The CR, which measures the reliability of pairwise comparisons, was calculated at 7.5%, remaining below the acceptable threshold of 0.10 (10%) as recommended by Saaty [20]. The AHP decision matrix is presented in Multimedia Appendix 1.

According to the results, visual clarity was ranked as the highest priority, receiving 36.4% of the total weight. This indicates that guidelines associated with visual clarity should take precedence, particularly in cases where they may conflict with other guidelines or functional aspects of e-tool development. Structure and navigation and ease of use were ranked second and third, with priority weights of 22.1% and 12.5%, respectively. A complete ranking of the principles and their corresponding weights is presented in Table 6.

**Table 6.** List of principles sorted by their rank and the percentage of their priority.

Principles	Rank	Priority, %
Visual clarity	1	36.4
Structure and navigation	2	22.1
Ease of use	3	12.5
Information	4	10.5
Minimizing the memory load	5	6.3
Feedback	6	3.8
Accessibility	7	2.8
Consistency	8	2.1
Simplicity	9	1.9
Control	10	1.8

## Discussion

### Principal Findings

This study aimed to identify, synthesize, and prioritize age-friendly usability design principles and guidelines for transportation planning e-tools tailored to the needs of older adults. Through a comprehensive scoping review and the application of the AHP, the study provides a structured, evidence-based framework to guide the design of inclusive digital transport tools. The synthesis of existing usability guidelines identified 68 clear and actionable recommendations, which were categorized under 10 core design principles. Among these, visual clarity, structure and navigation, and ease of use emerged as the highest priorities for enhancing the usability and comfort of digital tools for this population.

In general, our results highlighted the effectiveness of these generic usability guidelines in categorizing design principles tailored to specific user groups, particularly older adults. As Ahmad et al [59] pointed out, these generic usability guidelines function as essential rules of thumb that all user interface designers should follow. Although general guidelines are widely applicable, there is significant value in customizing them to meet the specific needs of older adults when developing age-friendly e-tools. Through actionable recommendations, this customization enhances the usability and adaptability of such e-tools. This perspective aligns with Patsoule and Koutsabasis [60], who argued that identifying and validating a set of principles and guidelines for web design specifically for older adults improves the adaptability of an e-tool to their needs and preferences.

More specifically, our results revealed that the principle of visual clarity was ranked as the highest priority, followed by structure and navigation and ease of use, which were ranked second and third, respectively. This underscores the critical importance of visual clarity in designing e-tools for older adults, particularly for travel planning, which is inherently complex and involves managing multiple types of information such as destinations, schedules, and personal preferences. A clear and intuitive interface reduces cognitive load, making tasks more manageable and less stressful, thereby improving usability and encouraging long-term adoption among older adults [61]. In support of these findings, Patsoule and Koutsabasis [60] highlighted “Visibility” and “Ease of Understanding” as the top 2 principles in age-friendly heuristics for redesigning websites for older adults. Their research aligns with the prioritization identified through the AHP methodology, further validating the emphasis on visual clarity, structure and navigation, and ease of use. These principles are vital in ensuring that older adults can effectively use e-tools without confusion or frustration.

Structure and navigation, ranked second (22.1%), further emphasizes the importance of logical content organization and user orientation within interfaces. Well-defined pathways, minimized data entry steps, and effective wayfinding features are essential to mitigate disorientation and cognitive overload [12,62]. Similarly, the third-ranked principle, ease of use (12.5%), points to the necessity of designing interfaces that are both intuitive and forgiving, offering support mechanisms such as autofill, orientation flexibility, and minimal setup requirements. At the other end of the spectrum, principles such as Control, Simplicity, and Consistency received the lowest prioritization. While their lower scores suggest they may not be as immediately critical as visual clarity or navigability, they remain essential for ensuring a robust and holistic user experience. To support this, Nahm et al [63] highlighted that “simple design with clear instructions” was a key preference among older adults, in contrast to design elements that rely on complex navigation or ambiguous features.

Given the dynamic nature of technology and the evolving needs of older adults, it is crucial to continuously refine and expand age-friendly usability design principles and guidelines. Nurgalieva et al [64] highlight that while many such guidelines exist, their adoption is often limited without ongoing efforts to make them more accessible, organized, and actionable. Furthermore, a recent systematic review of mobile app design for older adults emphasizes the importance of iterative, user-centered design, including feedback loops, adaptation to emerging devices, and integration of new accessibility features, to ensure that e-tools remain intuitive, usable, and relevant over time [65].

### Limitations

Although the study highlights the most relevant age-friendly usability design principles and guidelines for transportation planning e-tools, certain limitations should be acknowledged. First, the protocol for the scoping review was not registered in advance. Although the review followed established methodological frameworks and incorporated rigorous processes for data collection and analysis, the absence of a registered protocol may limit the transparency and reproducibility of the review process. To mitigate this limitation, steps were taken to enhance transparency and minimize bias: a second reviewer validated 20% of the study selection and data extraction, the search strategy was fully documented, and a professional librarian reviewed and refined the search approach. Notably, the methodology did not include the consultation step recommended by Levac et al [66], which is often used to enhance the relevance and applicability of findings through stakeholder engagement. Nevertheless, the results of this study were extensively applied in the Mobilaînés project [67] to support the co-design and development of an age-friendly transportation planning e-tool. The implementation of this innovative e-tool helped validate the practical value and effectiveness of the guidelines identified through the scoping review in addressing the digital accessibility needs of older adults. Second, although the data selection and extraction processes were confirmed by co-authors, study screening was performed by the first author. The validation process, while helpful, might not have fully addressed all possible sources of subjective influence. Future research could benefit from involving multiple reviewers in the data extraction and study selection stages, thereby providing a more balanced perspective and reducing the risk of individual biases affecting the outcomes. Third, the current study relied on a single expert for the AHP analysis, which may limit the generalizability and robustness of the prioritization results. To reduce this limitation, frequency data from the scoping review were integrated into the pairwise comparisons to provide a more objective basis alongside expert judgment, thereby helping to reduce potential bias. Nevertheless, involving multiple experts in future AHP analyses could further strengthen the validity of the findings. Incorporating diverse viewpoints and ensuring thorough cross-checking of the data would enhance the robustness and credibility of the review results. Fourth, it is possible that some articles were not retrieved, due to the selected keywords and lack of consideration for gray literature. Although the database searches included a broad range of academic publication types (eg, journal articles, conference papers, and proceedings), many age-friendly usability guidelines and design frameworks are disseminated through gray literature such as institutional reports, professional guidelines, and practitioner-oriented documents, which are not consistently indexed in bibliographic databases. To ensure comprehensive coverage, Google Scholar and Google Search were therefore used to identify gray literature, with the first 50 results from each platform screened to balance systematic coverage with feasibility. This complementary approach strengthened the inclusiveness of the review while acknowledging the inherent limitations of gray literature searching. Finally, while the search strategy may not have captured every possible variation in spelling, pluralization, or word order, the integration of standardized indexing terms (eg, MeSH) and a wide range of keyword formats enhanced the comprehensiveness of the literature search (Multimedia Appendix 2). The review and input from the professional librarian also played a key role in ensuring methodological soundness and adherence to systematic review guidelines.

### Conclusions

This study offers valuable insights into the design of transportation planning e-tools tailored to the needs of older adults, providing a robust foundation for the development of accessible and user-friendly digital interfaces. Indeed, the insights generated from this research are highly transferable, offering valuable contributions not only to transportation e-tools but also potentially to other apps and websites that support the autonomy and social participation of older adults. Continued refinement of these guidelines, through iterative updates and the active involvement of older users in the design process, will be crucial to ensure their long-term relevance and impact.

## Supplementary material

10.2196/83668 Multimedia Appendix 1 Analytic heirarchy process decision matrix.

10.2196/83668 Multimedia Appendix 2 Full search query example for MEDLINE.

10.2196/83668 Checklist 1 PRISMA-ScR checklist.

## Abbreviations

AHP: analytic hierarchy process
CI: consistency index
CR: consistency ratio
MeSH: Medical Subject Headings
PCC: population, concept, context
PRESS: Peer Review of Electronic Search Strategies
PRISMA-ScR: Preferred Reporting Items for Systematic Reviews and Meta-Analyses Extension for Scoping Reviews
RI: random index

## References

[1] Tomida K, Shimoda T, Nakajima C, Kawakami A, Shimada H (2024). Social isolation/loneliness and mobility disability among older adults. Curr Geri Rep.

[2] Che Had NH, Alavi K, Md Akhir N, Muhammad Nur IR, Shuhaimi MSZ, Foong HF (2023). A scoping review of the factor associated with older adults’ mobility barriers. Int J Environ Res Public Health.

[3] Andersen GJ (2012). Aging and vision: changes in function and performance from optics to perception. Wiley Interdiscip Rev Cogn Sci.

[4] Robnett RH, Chop WC (2013). Gerontology for the Health Care Professional.

[5] Ring L, Barry B, Totzke K, Bickmore T Addressing loneliness and isolation in older adults: proactive affective agents provide better support.

[6] Moyen de transport le plus couramment utilisé [Article in French]. Institut de la statistique du Québec.

[7] Bayne A, Siegfried A, Beck LF, Freund K (2021). Barriers and facilitators of older adults’ use of ride share services. J Transp Health.

[8] Holt-Lunstad J, Smith TB, Baker M, Harris T, Stephenson D (2015). Loneliness and social isolation as risk factors for mortality: a meta-analytic review. Perspect Psychol Sci.

[9] Xu R, Zhang S, Xiong P, Lin AY, Ma J Towards better driver safety: empowering personal navigation technologies with road safety awareness.

[10] Whiteside MM, Wallhagen MI, Pettengill E (2006). Sensory impairment in older adults: part 2: vision loss. Am J Nurs.

[11] Dixon J, Crooks H, Henry K (2006). Breaking the ice: supporting collaboration and the development of community online. Can J Learn Technol.

[12] Murman DL (2015). The impact of age on cognition. Semin Hear.

[13] Dodd C, Athauda R, Adam M Designing user interfaces for the elderly: a systematic literature review. https://aisel.aisnet.org/acis2017/61.

[14] Bahrampoor Givi S, Gagnon-Roy M, Pigot H, Provencher V (2025). User experience of older adults with an age‑friendly transportation planning e‑tool: scoping review. JMIR Hum Factors.

[15] Nielsen J (1994). Usability Engineering.

[16] Jiang Q, Deng L, Zhang J, Pengbo Y (2024). User-centered design strategies for age-friendly mobile news apps. SAGE Open.

[17] Zaphiris P, Ghiawadwala M, Mughal S Age-centered research-based web design guidelines.

[18] Arksey H, O’Malley L (2005). Scoping studies: towards a methodological framework. Int J Soc Res Methodol.

[19] Tricco AC, Lillie E, Zarin W (2018). PRISMA Extension for Scoping Reviews (PRISMA‑ScR): checklist and explanation. Ann Intern Med.

[20] Saaty TL (2008). Decision making with the analytic hierarchy process. Int J Serv Sci.

[21] McGowan J, Sampson M, Salzwedel DM, Cogo E, Foerster V, Lefebvre C (2016). PRESS Peer Review of Electronic Search Strategies: 2015 guideline statement. J Clin Epidemiol.

[22] Shneiderman B, Plaisant C (2004). Designing the user interface: strategies for effective human‑computer interaction.

[23] Gerhardt‐Powals J (1996). Cognitive engineering principles for enhancing human‐computer performance. Int J Hum Comput Interact.

[24] Weinschenk S, Barker DT (2000). Designing Effective Speech Interfaces.

[25] Braun V, Clarke V (2006). Using thematic analysis in psychology. Qual Res Psychol.

[26] Saaty TL (2012). Decision Making for Leaders: The Analytic Hierarchy Process for Decisions in a Complex World.

[27] Goepel KD (2018). Implementation of an online software tool for the Analytic Hierarchy Process (AHP‑OS). Int J Analytic Hierarchy Process.

[28] Abdullah N, Abdul Hamid NFB (2019). Interface design features of mobile application for senior citizens. Indones J Elect Eng Comput Sci.

[29] Alkhomsan MN, Alturayeif N, Alwadei S, Baslyman M (2023). UsAge guidelines: toward usable Saudi M-Government applications for elderly users. J King Saud Univ Comput Inf Sci.

[30] (2019). Accessibility. Material Design.

[31] Barros A de, Leitão R, Ribeiro J Design and evaluation of a mobile user interface for older adults: navigation, interaction and visual design recommendations.

[32] Bong WK, Bronshtein I Designing digital games with & for home-dwelling older adults’ social interaction under sheltering measures.

[33] (2024). UX/UI design for elderly users: best practices & principles. LinkedIn.

[34] Dombrovskaia L, Vilches A Guideline for web pages design inclusive for senior users.

[35] Finn K, Johnson J Designing for an aging population: toward universal design.

[36] Friedman V (2024). A guide to designing for older adults. Smashing Magazine.

[37] Gao Q, Sun Q (2015). Examining the usability of touch screen gestures for older and younger adults. Hum Factors.

[38] Ghorbel F, Métais E, Ellouze N, Hamdi F, Gargouri F Towards accessibility guidelines of interaction and user interface design for alzheimer’s disease patients. https://personales.upv.es/thinkmind/dl/conferences/achi/achi_2017/achi_2017_6_30_20152.pdf.

[39] Gomez-Hernandez M, Ferre X, Moral C, Villalba-Mora E (2023). Design guidelines of mobile apps for older adults: systematic review and thematic analysis. JMIR mHealth uHealth.

[40] Gruver J (2024). UX/UI design for elderly users: a comprehensive guide. Bootcamp (Medium).

[41] Halperin Ben Zvi M, Briscoe G, Sidse C Age inclusive digital platforms. https://researchonline.rca.ac.uk/5207/.

[42] Lum ASL, Chiew TK, Ng CJ, Lee YK, Lee PY, Teo CH (2017). Development of a web-based insulin decision aid for the elderly: usability barriers and guidelines. Univ Access Inf Soc.

[43] Morey SA, Stuck RE, Chong AW, Barg-Walkow LH, Mitzner TL, Rogers WA (2019). Mobile health apps: improving usability for older adult users. Ergonom Des.

[44] Nasr E, Alsaggaf W, Sinnari D (2023). Developing Usability Guidelines for mHealth Applications (UGmHA). Multimodal Technol Interact.

[45] (2001). Making your web site senior friendly: a checklist. https://www.huzurevleri.org.tr/docs/MakingYourWebsiteSeniorFriendly.pdf.

[46] Panagopoulos C, Menychtas A, Tsanakas P, Maglogiannis I (2019). Increasing usability of homecare applications for older adults: a case study. Designs.

[47] Patch K, Spellman J, Wahlbin K (2015). Mobile accessibility: how WCAG 2.0 and other W3C/WAI guidelines apply to mobile. World Wide Web Consortium (W3C).

[48] Petrovčič A, Taipale S, Rogelj A, Dolničar V (2018). Design of mobile phones for older adults : an empirical analysis of design guidelines and checklists for feature phones and smartphones. Int J Hum–Comput Interact.

[49] Rosman S, Siau NZ, Bramantoro A Evaluating user interface and user experience in mobile applications designed for the elderly.

[50] Ruzic L, Lee ST, Liu YE, Sanford JA, Antona M, Stephanidis C Development of universal design mobile interface guidelines (UDMIG) for aging population.

[51] Sakdulyatham R, Preeyanont S, Lipikorn R, Watakakosol R (2017). User interface on smartphone for elderly users. Int J Autom Smart Technol.

[52] Vasudavan H, Jayabalan M, Ramiah S (2015). A preliminary study on designing tour website for older people.

[53] Zahida W, Effendy V, Hadikusuma A Improving messenger accessibility for elderly users using user centered design (UCD) methods (study case: WhatsApp).

[54] A guide to interface design for older adults. Adchitects.

[55] Polyuk S Age before beauty – a guide to interface design for older adults. Toptal.

[56] (2016). FACTSHEET on SS 618 – guidelines on user interface design for older adults. https://www.nas.gov.sg/archivesonline/data/pdfdoc/20170323006/Media%20Factsheet_%20Silver%20Industry%20Roadmap%20and%20SS%20618%20Launch_FINAL.pdf.

[57] Designing for iOS. Apple Developer Documentation.

[58] (2022). UI design for elderly. UserPeek.

[59] Ahmad N, Rextin A, Kulsoom UE (2018). Perspectives on usability guidelines for smartphone applications: an empirical investigation and systematic literature review. Inf Softw Technol.

[60] Patsoule E, Koutsabasis P (2014). Redesigning websites for older adults: a case study. Behav Inf Technol.

[61] Zhang X, Dolah J (2024). A study of key factors influencing the usability of smartphone graphical user interfaces for older adults. PaperASIA.

[62] Zhou C, Yuan F, Huang T, Zhang Y, Kaner J (2022). The impact of interface design element features on task performance in older adults: evidence from eye-tracking and EEG signals. Int J Environ Res Public Health.

[63] Nahm ES, Preece J, Resnick B, Mills ME (2004). Usability of health web sites for older adults: a preliminary study. Comput Inform Nurs.

[64] Nurgalieva L, Jara Laconich JJ, Baez M, Casati F, Marchese M (2019). A systematic literature review of research-derived touchscreen design guidelines for older adults. IEEE Access.

[65] Amouzadeh E, Dianat I, Faradmal J, Babamiri M (2025). Optimizing mobile app design for older adults: systematic review of age-friendly design. Aging Clin Exp Res.

[66] Levac D, Colquhoun H, O’Brien KK (2010). Scoping studies: advancing the methodology. Implement Sci.

[67] Provencher V, Baillargeon D, Abdulrazak B (2022). Developing a one-stop platform transportation planning service to help older adults move around in their community where, when, and how they wish: protocol for a living lab study. JMIR Res Protoc.

