# Supplementary material for: Identifying and Prioritizing Age-Friendly Design Principles and Guidelines for Developing Transportation Planning E-Tools: Scoping Review
Source: JMIR Aging. 2026 Mar 19;9:e83668. doi: 10.2196/83668 (PMC13002004; doi:10.2196/83668)
Supplement: Multimedia Appendix 1 [file aging-v9-e83668-s001.docx]

**Appendix 1: AHP Decision Matrix**

| **Principles** |  | **Feedback** | **Consistency** | **Minimizing the Memory Load** | **Simplicity** | **Accessibility** | **Structure & Navigation** | **Control** | **Ease of Use** | **Visual Clarity** | **Information** |
| --- | --- | --- | --- | --- | --- | --- | --- | --- | --- | --- | --- |
| **Feedback** |  | 1.00 | 3.00 | 0.33 | 3.00 | 2.00 | 0.17 | 3.00 | 0.20 | 0.13 | 0.25 |
| **Consistency** |  | 0.33 | 1.00 | 0.20 | 1.00 | 0.33 | 0.13 | 2.00 | 0.20 | 0.11 | 0.25 |
| **Minimizing the Memory Load** |  | 3.00 | 5.00 | 1.00 | 4.00 | 4.00 | 0.17 | 4.00 | 0.25 | 0.13 | 0.33 |
| **Simplicity** |  | 0.33 | 1.00 | 0.25 | 1.00 | 0.50 | 0.13 | 1.00 | 0.17 | 0.11 | 0.20 |
| **Accessibility** |  | 0.50 | 3.00 | 0.25 | 2.00 | 1.00 | 0.13 | 2.00 | 0.17 | 0.11 | 0.14 |
| **Structure & Navigation** |  | 6.00 | 8.00 | 6.00 | 8.00 | 8.00 | 1.00 | 7.00 | 4.00 | 0.33 | 3.00 |
| **Control** |  | 0.33 | 0.50 | 0.25 | 1.00 | 0.50 | 0.14 | 1.00 | 0.17 | 0.11 | 0.14 |
| **Ease of Use** |  | 5.00 | 5.00 | 4.00 | 6.00 | 6.00 | 0.25 | 6.00 | 1.00 | 0.14 | 2.00 |
| **Visual Clarity** |  | 8.00 | 9.00 | 8.00 | 9.00 | 9.00 | 3.00 | 9.00 | 7.00 | 1.00 | 5.00 |
| **Information** |  | 4.00 | 4.00 | 3.00 | 5.00 | 7.00 | 0.33 | 7.00 | 0.50 | 0.20 | 1.00 |

| **Principles** | **Priority** | **Rank** | **(+)** | **(-)** |
| --- | --- | --- | --- | --- |
| Feedback | 3.8% | 6 | 1.4% | 1.4% |
| Consistency | 2.1% | 8 | 0.9% | 0.9% |
| Minimizing the Memory Load | 6.3% | 5 | 2.9% | 2.9% |
| Simplicity | 1.9% | 9 | 0.7% | 0.7% |
| Accessibility | 2.8% | 7 | 1.3% | 1.3% |
| Structure & Navigation | 22.1% | 2 | 11.0% | 11.0% |
| Control | 1.8% | 10 | 0.8% | 0.8% |
| Ease of Use | 12.5% | 3 | 5.9% | 5.9% |
| Visual Clarity | 36.4% | 1 | 21.6% | 21.6% |
| Information | 10.5% | 4 | 4.4% | 4.4% |
