# Supplementary material for: Identifying and Prioritizing Age-Friendly Design Principles and Guidelines for Developing Transportation Planning E-Tools: Scoping Review
Source: JMIR Aging. 2026 Mar 19;9:e83668. doi: 10.2196/83668 (PMC13002004; doi:10.2196/83668)
Supplement: Multimedia Appendix 2 [file aging-v9-e83668-s002.docx]

**Full Search Query Example for MEDLINE (PubMed, NCBI)**

To combine the concepts and keywords, the following search strategy was used in MEDLINE (PubMed):

**Concept 1: Aging**

("older adult*" OR "old people" OR elderly OR senior* OR aging OR ageing OR "normal aging" OR frailty OR aged OR old* OR Aged[MeSH])

**AND**

**Concept 2: E-tool**

(website* OR application* OR "e-tool" OR "web application*" OR app* OR web OR Mobile Applications[MeSH])

**AND**

**Concept 3: Guideline**

("design principle*" OR "web design principle*" OR "design guideline*" OR "web design guideline*" OR "usability guideline*" OR "user interface design guideline*" OR "UI design guideline*" OR "user experience design guideline*" OR "UX design guideline*" OR GUI OR "graphical user interface" OR User-Computer Interface[MeSH] OR Guidelines as Topic[MeSH])

**AND**

**Concept 4: Transport**

(transport* OR "public transport*" OR mobility* OR travel OR "public transit" OR "active transport*" OR "alternative transport" OR paratransit OR bus* OR carpool* OR Transportation[MeSH])

**Search Limits and Filters**

Language: English

Date Range: From January 2013 – May 2023

Updated April 2024

Updated April 2024
